# Effects of visible implanted elastomer marking on physiological traits of frogs

**DOI:** 10.1093/conphys/cou042

**Published:** 2014-10-03

**Authors:** Rachael E. Antwis, Rebecca Purcell, Susan L. Walker, Andrea L. Fidgett, Richard F. Preziosi

**Affiliations:** 1 Faculty of Life Sciences, University of Manchester, Carys Bannister Building, Dover Street, Manchester M13 9PL, UK; 2 Chester Zoo, Upton-by-Chester, Chester CH2 1EU, UK

**Keywords:** Amphibian, glucocorticoid, marking technique, peptide, symbiotic bacteria

## Abstract

Amphibians possess innate immune defences, including antimicrobial peptides and symbiotic bacterial communities, that can protect them from infectious diseases, including chytridiomycosis. On-going research is attempting to use amphibian symbiotic bacteria to develop probiotic treatments that can protect hosts from the causative agent of chytridiomycosis, the fungal pathogen *Batrachochytrium dendrobatidis*. Events that cause disruption of symbiotic bacterial communities or deplete peptide stores could increase the susceptibility of individuals to disease and may have implications for amphibians involved in probiotic trials or time course studies that investigate symbiotic bacterial communities. It has previously been shown that passive integrated transponder tagging of frogs causes a rapid (within 24 h) and major proliferation of micro-organisms on the skin. Here, we show that marking of red-eyed tree frogs (*Agalychnis callidryas*) with visible elastomer has no effect on adrenal response (represented by faecal glucocorticoid metabolite concentrations) or peptide production, although there was evidence of a slightly greater microbial abundance associated with the skin of marked frogs 2 weeks after tagging. The results indicate that visible elastomer may be a preferable marking technique to passive integrated transponder tagging, particularly in the context of probiotic trials or time course studies that investigate symbiotic bacterial communities. More work is required to determine the effects of different marking techniques on physiological responses of amphibians, whether these physiological responses are consistent across host species and whether such ‘non-invasive’ marking methods affect the susceptibility of amphibians to infectious pathogens, such as *B. dendrobatidis*.

## Abstract (precis)

Proteins and bacteria on the skin of amphibians can protect them from diseases. Marking frogs with elastomer tags had no effect on stress hormone production or protein release, but lead to a slightly greater abundance of skin microbes. This marking technique may be a comparatively safe option for amphibians.

## Introduction

Amphibians are currently undergoing major global population declines, a major driver of which are emerging infectious diseases, such as chytridiomycosis and ranaviruses (reviewed by Gray *et al.*, 2009; Kilpatrick *et al.*, 2010). Amphibians possess innate immune defences, including antimicrobial peptides and symbiotic bacterial communities, that can protect them from such diseases (reviewed by Rollins-Smith and Conlon, 2005; Rollins-Smith *et al.*, 2011; Bletz *et al.*, 2013). The potential for using symbiotic bacteria for probiotic treatments against chytridiomycosis is currently being investigated (reviewed by Bletz *et al.*, 2013), and on-going research has identified symbiotic bacteria that inhibit the growth of the chytridiomycosis fungus, *Batrachochytrium dendrobatidis*, from a number of amphibian species (e.g. Harris *et al.*, 2006; Culp *et al.*, 2007; Lauer *et al.*, 2007; Banning *et al.*, 2008; Brucker *et al.*, 2008; Flechas *et al.*, 2012; Loudon *et al.*, 2013; Roth *et al.*, 2013).

Events that cause disruption of symbiotic bacterial communities or deplete peptide stores could potentially increase the susceptibility of individuals to disease and may have implications for amphibians involved in probiotic trials or time course studies that investigate symbiotic bacterial communities. Wild and captive amphibians are often marked for a variety of reasons, including identification of individuals, in order to avoid resampling, or to conduct mark–release–recapture surveys. Historically, toe-clipping was often used for identification, although less invasive techniques, such as passive integrated transponder (PIT) tagging or visible implant elastomer (VIE) dyes, are now more commonly used. The existing literature indicates that these marking techniques have few negative effects on amphibians (e.g. Kinkead *et al.*, 2006; Phillips and Fries, 2009; Schmidt and Schwarzkopf, 2010; Sapsford *et al.*, 2014), although a recent study showed that PIT tagging causes a proliferation in resident bacteria and fungi on the skin of frogs (Antwis *et al.*, 2014a).

Amphibian peptides are produced and stored in granular glands and control the colonization of the skin by both pathogenic and symbiotic micro-organisms (reviewed by Conlon, 2011). It is thought that amphibian peptides have a reciprocal relationship with symbiotic bacterial communities on the skin and may play a role in maintaining the bacterial community by limiting their antimicrobial potency (reviewed by Boman, 2000; Conlon, 2011). Although peptide production and secretion occurs continuously at a low level, it increases substantially in response to stressful events, such as alarm, injury or pathogenic infection (Rollins-Smith *et al.*, 2011; Pask *et al.*, 2012). Peptide granular glands are surrounded by a layer of myoepithelial cells containing α-adrenoreceptors that are activated by adrenaline (epinephrine) or noradrenaline (norepinephrine) to induce the release of peptides (reviewed by Rollins-Smith, 2001; Delfino *et al.*, 2006). The greater the stress experienced, the greater the level of peptide release by an individual; *Xenopus laevis* injected with increasing concentrations of noradrenaline showed a concurrent increase in release of peptides (Rollins-Smith *et al.*, 2005). Particularly stressful events can cause granular glands to become exhausted of peptides, and studies have shown that concurrent increases in glucocorticoids lead to the inhibition of transcription factors required to produce peptides (Simmaco *et al.*, 1997, 1998). This temporary absence of peptides can lead to the proliferation of bacterial communities (Simmaco *et al.*, 1998) and may explain the results seen by Antwis *et al.* (2014a).

There are limited studies that have investigated the effects of marking techniques on steroid (e.g. adrenaline, noradrenaline, glucocorticoid) production in amphibians. Kinkead *et al.* (2006) showed that two species of salamanders (*Desmognathus fuscus* and *Desmognathus monticola*) marked with VIE or toe-clipped under varying degrees of anaesthesia did not exhibit increased noradrenalineor adrenaline levels in comparison to unmarked salamanders. Handling has been shown to increase circulating glucocorticoid concentrations in a range of amphibian species (Woodley and Lacy, 2010; Narayan *et al.*, 2011a, 2012a, 2013), and toe-clipping causes a significant and prolonged (>3 days) increase in circulating corticosterone in cane toads (*Rhinella marina*; Narayan *et al.*, 2011b). However, to the authors' knowledge, the effects of other marking techniques on glucocorticoid production have not been evaluated for amphibians, along with concurrent effects on peptide release and bacterial communities. Here, we determine the effects of VIE marking on culturable cutaneous microbial abundance, peptide release and faecal glucocorticoid metabolite concentrations of captive red-eyed tree frogs (*Agalychnis callidryas*).

## Materials and methods

### Ethics statement

The Chester Zoo Ethical Committee and the University of Manchester Ethics Committee approved this study prior to starting. This study did not require a UK Home Office licence because VIE tagging is an approved method for the identification of frogs, and data were collected during routine marking of frogs for identification purposes. Frogs were checked daily throughout the study and for 2 weeks after for any signs of adverse reaction to any of the techniques used, none of which was observed.

### Study animals, husbandry and experimental design

A total of 16 adult *A. callidryas* were used in this study; eight (four males, four females) in the control (unmarked) group and eight (four males, four females) in the marked group. All frogs were from the same clutch of F3 generation captive-bred individuals and had not previously been involved in any other studies. Prior to the start of the experiment, frogs were group housed in 60 × 45 × 45 cm glass tanks (ExoTerra^®^, UK) with a naturalistic set-up consisting of a LECA base for drainage and a soil layer covered with dried oak leaves, and planted with a peace lily and cuttings of devil's ivy. Two weeks prior to the start of the study, frogs were randomly assigned to 16 individual experimental housing tubs (30 × 18 × 20 cm ExoTerra^®^ plastic faunariums), with frogs from the same group housed tanks spread across treatment groups to allow for prior variation in bacterial communities. Individual housing was required to distinguish faecal samples between individuals and to avoid the potential for bacterial communities to be confounded by group housing during the study. Experimental tanks were lined with damp paper towels and contained a water dish and a cutting of devil's ivy. Paper towels and water dishes were changed twice weekly. All tanks were maintained with a ZooMed Reptisun^®^ 10.0 ultraviolet strip light with reflectors and a Philips daylight bulb with reflectors, both of which were on a 10:14 light:dark cycle. Frogs were fed three or four times weekly with black crickets (*Gryllus bimaculatus*) gut-loaded on fresh fruit and vegetables and dusted with Nutrobal (VetArk, UK).

After a 2 week habituation period in the individual housing, faecal samples were collected daily from day 1 until the end of the study (day 28) to measure faecal glucocorticoid metabolite concentrations (see ‘*Faecal glucocorticoid metabolite concentrations*’ section below for further details). On day 8, frogs were sampled for bacterial communities and peptide release as described below (see ‘*Microbial communities*’ and ‘*Peptide release*’ sections). This provided baseline data for each individual, which is referred to hereafter as ‘start of study’. On day 14, frogs in the ‘marked’ group were tagged using VIE dye (see ‘*Marking techniques*’ below for details). On day 15, all frogs were sampled again for bacterial communities and peptide release, and these data are referred to as ‘post-marking’. On day 28, bacterial communities and peptide release were sampled again, and these data are referred to as ‘end of study’.

### Marking techniques

*Agalychnis callidryas* in the marked group were tagged on day 14 of the study using VIE dye (Northwest Marine Technology Inc., USA). This method was chosen because frogs were due to be rehomed at another institution and it was necessary to distinguish these frogs from existing *A. callidryas* in the collection at the receiving institution without the requirement for individual identification. The elastomer dye was prepared according to the manufacturer's instructions. Gloves were worn throughout marking and changed between frogs to minimize cross-contamination. During marking, the frog was restrained on a flat and stable surface that had previously been sterilized, and the sterile needle containing the dye was inserted smoothly and quickly under the skin of the underside of the tibiofibular (G. Garcia, personal communication). The marking process was standardized and took ∼30 s per individual. In order to determine whether the whole marking process affected physiological traits of frogs, control frogs were not sham injected or manipulated at the post-marking time point. All frogs were monitored for the remainder of the study and for 2 weeks after the end of the study for any signs of adverse reaction, none of which was observed.

### Faecal glucocorticoid metabolite concentrations

Tanks were checked daily within an hour of the lights coming on (*A. callidryas* are nocturnal and so defaecate at night), and any faecal samples were frozen at −20°C for subsequent analyses. Gloves were used to handle samples and changed between to avoid contamination. Glucocorticoid metabolite analyses were conducted at the Chester Zoo's Wildlife Endocrinology Laboratory.

Glucocorticoid metabolites were extracted from faecal samples following thawing and manual homogenization using a wet-weight shaking extraction adapted from Walker *et al.* (2002). In brief, the individual faecal sample weight was recorded (mean = 0.138 ± 0.007 g) and then combined with 90% methanol, shaken overnight at room temperature and centrifuged for 20 min at 598***g***. The supernatant was decanted and evaporated to dryness under air. Faecal extracts were resuspended in 0.25 ml of 100% methanol and stored at −20°C until analysis.

Glucocorticoid metabolites (corticosterone) were analysed using a modified enzyme immunoassay (EIA) as previously described (Munro and Stabenfeldt, 1984; Watson *et al.*, 2013). Each EIA used an antibody (polyclonal corticosterone antiserum CJM006 supplied by C. J. Munro, University of California, Davis, CA, USA), horseradish peroxidase-conjugated label (corticosterone prepared according to Munro and Stabenfeldt, 1984) and standards (corticosterone; Sigma-Aldrich, UK). The modified assay procedures were as follows for the corticosterone EIA. Antiserum was diluted at 1:15 000 in coating buffer (0.05 m NaHCO_3_, pH 9.6), and 50 μl per well was loaded onto a 96-well Nunc-Immuno Maxisorp (Thermo-Fisher Scientific) microtiter plate, which was covered with a plate sealer and left overnight at 4°C. Plates were washed five times (0.15 m NaCl, 0.05% Tween 20) and then 50 μl per well of each standard and sample (3.9–1000 pg per well or samples diluted 1:10 in EIA buffer) were loaded in duplicate with 50 μl per well of horseradish peroxidase conjugate diluted in EIA buffer (1:70 000). Following incubation in the dark for 2 h at room temperature, plates were washed five times and incubated with 100 μl per well of substrate [0.4 mm 2,2′-azino-di-(3-ethylbenzthiazoline sulfonic acid) diammonium salt, 1.6 mm H_2_O_2_ and 0.05 m citrate, pH 4.0], left to develop at room temperature in the dark and measured at 405 nm at optical density 0.8–1.0. The corticosterone EIA was biochemically validated for measuring corticosterone metabolites in *A. callidryas* faeces through parallelism (sample percentage binding = 30.606 ± 0.8129, *r*^2^ = 0.9209, *F*_1,7_ = 81.461, *P* < 0.001) and matrix interference assessment (observed = 31.074 ± 1.522, *r*^2^ = 0.998, *F*_1,7_ = 4089.8, *P* < 0.001). The cross-reactivities for corticosterone antiserum CJM006 have been reported elsewhere (Watson *et al.*, 2013), and the intra- and interassay variation were 7.52 and 6.33%, respectively. The assay was biologically validated by demonstrating a significant rise in faecal glucocorticoid metabolite concentrations from prior to any manipulations being conducted on frogs (i.e. days 1–8) to the highest value collected for each individual after this time (days 9–28; Student's paired *t* test; *t* = 3.213, d.f. = 15, *P* = 0.006).

**Figure 1:**
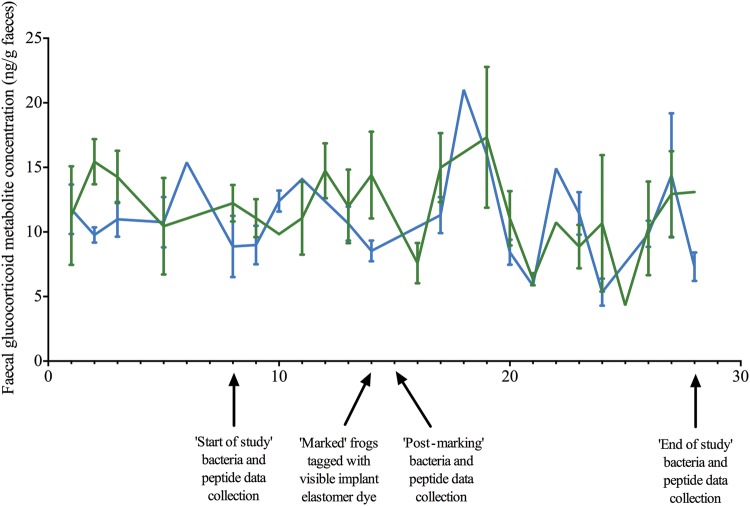
Faecal glucocorticoid metabolite concentrations for control (green) and visible implant elastomer dye (VIE)-marked *Agalychnis callidryas* (blue) over the 28 day study period. Error bars show ±1 SEM.

Final corticosterone metabolite concentrations were calculated per gram of wet faecal mass. Data collected before any handling or marking events (days 1–7) were assigned as ‘start of study’, data collected from 1 day post-marking until 5 days post-marking (days 16–19) were assigned as ‘post-marking’, and data collected from day 20 until day 28 were assigned as ‘end of study’. Raw data values for faecal glucocorticoid metabolite concentrations indicated an increase at around 3–5 days post-marking (days 17–19; Fig. 1), which is within the expected lag time in glucocorticoid metabolite deposition in faeces for a range of species (Wasser *et al.*, 2000; Bamberg *et al.*, 2001; Martínez-Mota *et al.*, 2008; Santymire *et al.*, 2012; Smith *et al.*, 2012) including amphibians (Cikanek *et al.*, 2014). Therefore these data were used for the post-marking time point as described above. Data were analysed in RStudio (using the lme4 package) for effects of treatment, time point and the interaction between treatment and time point using a generalized linear mixed model, with individual included as a random effect, with Tukey's *post hoc* analysis for pairwise comparisons.

### Microbial communities

Microbial communities were collected using similar methods to those previously described by Antwis *et al.* (2014b). Briefly, frogs were rinsed to remove transient bacteria using 50 ml of ultrapure water, which was retained for peptide quantification (see ‘*Peptide release*’ below). Frogs were then swabbed on their ventral surface 20 times using sterile swabs (Eurotubo^®^, Rubi, Spain). Gloves were worn throughout the swabbing process, which took ∼30 s per frog, and changed between individuals to minimize cross-contamination. Swabbing methods were consistent across individuals, treatment groups and time points. Swabs were placed into 1 m NaCl_2_ solution and serial dilutions constructed up to a concentration of 10^−2^. Concentrations of 10^−1^ and 10^−2^ were plated out on low-nutrient agar (R2A; Lab M, UK). Bacterial and fungal colony abundances were counted every 2 days until negligible new growth was observed (10 days after initial plating). Bacterial and fungal counts were then multiplied by their respective dilution factors and averaged for each individual. Data for bacterial abundance and fungal abundance were analysed separately in RStudio (using the lme4 package) for effects of treatment, time point and the interaction between treatment and time point using a generalized linear mixed model with Poisson distribution, with individual included as a random effect. Tukey's *post hoc* analysis was conducted for pairwise comparisons.

### Peptide release

Prior to sampling for cutaneous microbes, frogs were rinsed on their dorsal and ventral surfaces by pouring 50 ml of ultrapure water over each surface five times. The final volume of liquid collected was recorded, and peptides were extracted from the water using previously described techniques (Rollins-Smith *et al.*, 2002; Woodhams *et al.*, 2006; Sheafor *et al.*, 2008; Conlon *et al.*, 2011). Briefly, samples were acidified to 1% using trifluoroacetic acid (TFA; Sigma Aldrich, UK) and then passed over individual Sep-Pak C-18 cartridges (Waters Associates, Milford, MA, USA) at a rate of 5 ml/min. Sep-Pak cartridges were primed prior to use with 1 ml of 1% TFA solution as recommended by the manufacturers. Peptides were then eluted using 2 ml of elution solution composed of 70% acetonitrile (Sigma Aldrich, UK), 29.9% ultrapure water and 0.1% TFA. Eluted peptides were concentrated to dryness in a vacuum centrifuge (Genevac MiVac Modular Concentrator System) and reconstituted in 150 μl of ultrapure water.

Peptides were quantified in triplicate for each sample using a BCA Protein Assay Kit (Thermo Scientific Pierce, UK) according to the manufacturer's instructions, except that bradykinin (Sigma Aldrich, UK) was used to establish a standard curve (Rollins-Smith *et al.*, 2002; Woodhams *et al.*, 2006; Sheafor *et al.*, 2008; Conlon *et al.*, 2011). Interpolated peptide concentrations were averaged for each individual and adjusted by a scaling factor that accounted for the volume of liquid passed through the SepPak cartridges, and then divided by the mass of the corresponding frog to give a final peptide concentration per gram of frog. Peptide data were analysed in RStudio (using the lme4 package) for effects of treatment, time point and the interaction between treatment and time point using a generalized linear mixed model with Poisson distribution, with individual included as a random effect. Tukey's *post hoc* analysis was conducted for pairwise comparisons.

## Results

### Faecal glucocorticoid metabolite concentration

**Figure 2:**
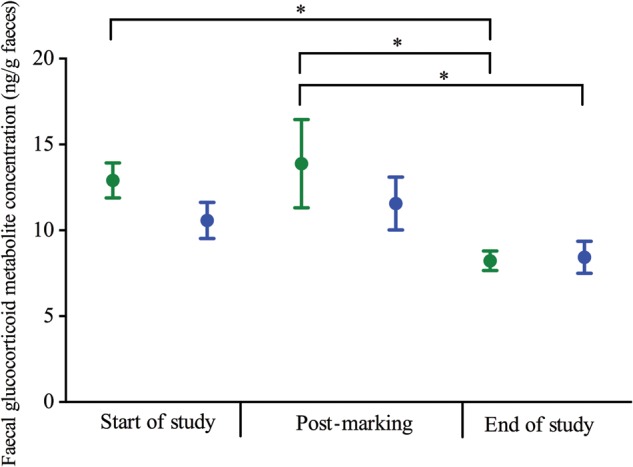
Average faecal glucocorticoid metabolite concentrations for control (green) and VIE-marked *A. callidryas* (blue) at the start of the study, post-marking and at the end of the study. Error bars show ±1 SEM. An asterisk indicates a significantly different result (*P* < 0.05).

There was a significant effect of time (χ^2^ = 29.679, d.f. = 2, *P* < 0.001) but not treatment (χ^2^ = 1.257, d.f. = 1, *P* = 0.0262) or the interaction between treatment and time (χ^2^ = 1.990, d.f. = 2, *P* = 0.370; Figs 1 and 2) on faecal glucocorticoid metabolite concentrations. *Post hoc* analyses showed significant differences between control frogs at the start of the study and at the end of the study (*P* = 0.002) and between control frogs post-marking and at the end of the study (*P* = 0.001), with end-of-study faecal glucocorticoid metabolite concentrations being lower at the end of the study than at the other two time points (Supplementary Table S1; Fig. 2). Marked frogs at the end of the study had significantly lower faecal glucocorticoid metabolite concentrations than control frogs post-marking (Supplementary Table S1; Fig. 2). As time was the only statistically significant predictor of faecal glucocorticoid metabolite concentrations in the original model, a simplified generalized linear mixed model containing only this variable was run. This model was statistically significant (χ^2^ = 29.282, d.f. = 2, *P* < 0.001), and *post hoc* analyses showed that faecal glucocorticoid metabolite concentrations at the end of the study were significantly lower than those at the start of the study (*P* < 0.001) and post-marking (*P* < 0.001) across all frogs, but there were no significant differences between faecal glucocorticoid metabolite concentrations at the start of the study and post-marking (*P* = 0.377).

### Microbial communities

**Figure 3:**
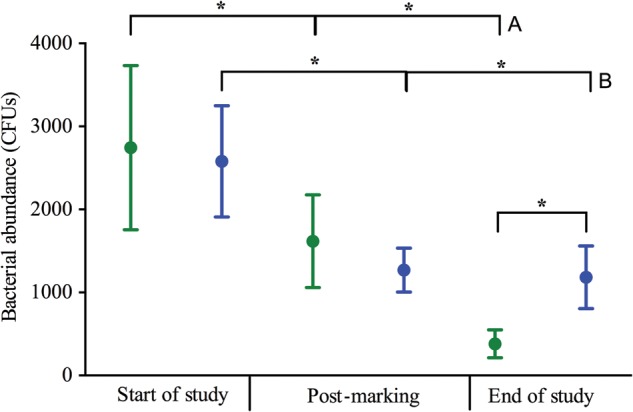
Average cutaneous bacterial abundance (in colony-forming units, CFUs) for control (green) and VIE-marked *A. callidryas* (blue) at the start of the study, post-marking and at the end of the study. Error bars show ±1 SEM. An asterisk indicates a significantly different result (*P* < 0.05; only main comparisons are shown, and all significant results are shown in Supplementary Table S1). Group A significance bars are all statistically different to one another and indicate a significant decrease in bacterial abundance over time for control frogs, and likewise for group B significance bars for marked frogs.

There was a significant effect of time (χ^2^ = 12 800, d.f. = 2, *P* < 0.001) and the interaction between treatment and time (χ^2^ = 2893, d.f. = 2, *P* < 0.001) on bacterial abundance, but not of treatment alone (χ^2^ = 0.963, d.f. = 1, *P* = 0.326). *Post hoc* analyses showed overall that there were significant decreases in bacterial abundance over time for both treatment groups, and the bacterial abundance associated with control frogs at the end of the study was significantly lower than that of marked frogs at all time points (Supplementary Table S1; Fig. 3). In particular, marked frogs had significantly greater bacterial abundance than control frogs at the end of the study (i.e. 2 weeks post-marking).

**Figure 4:**
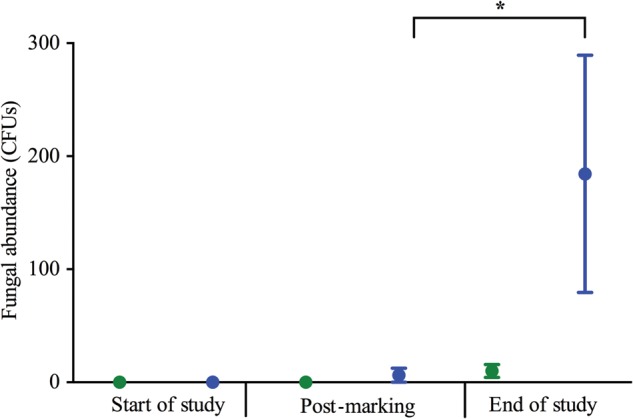
Average cutaneous fungal abundance for control (green) and VIE-marked *A. callidryas* (blue) at the start of the study, post-marking and at the end of the study. Error bars show ±1 SEM. An asterisk indicates a significantly different result (*P* < 0.05).

There was a significant effect of time (χ^2^ = 544, d.f. = 2, *P* < 0.001) on the abundance of fungi associated with the skin of frogs, but no significant effects of treatment (χ^2^ = 1.309, d.f. = 1, *P* = 0.253) or the interaction between time and treatment (χ^2^ = 0.016, d.f. = 2, *P* = 0.992). *Post hoc* analyses showed that marked frogs had a significantly greater fungal abundance at the end of the study than post-marking (Supplementary Table S1; Fig. 4).

### Peptide release

**Figure 5:**
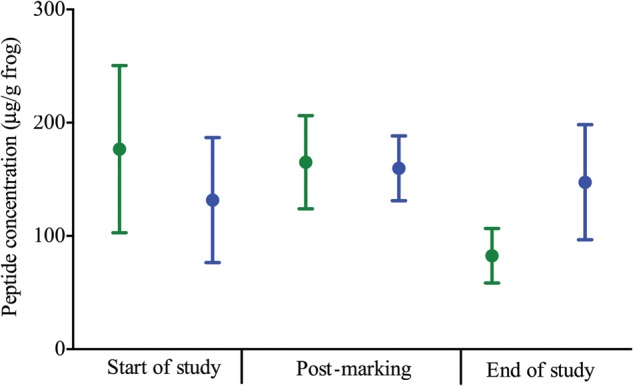
Average peptide concentrations for control (green) and VIE-marked *A. callidryas* (blue) at the start of the study, post-marking and at the end of the study.

There were no significant effects of time (χ^2^ = 2.226, d.f. = 2, *P* = 0.329), treatment (χ^2^ = 0.042, d.f. = 1, *P* = 0.838) or the interaction between treatment and time (χ^2^ = 2.531, d.f. = 2, *P* = 0.282) on the release of peptides by frogs (Fig. 5), and none of the pairwise comparisons was statistically significant (Supplementary Table S1).

## Discussion

In this study, we found no evidence that VIE marking of frogs leads to an increase in faecal glucocorticoid metabolite concentrations in comparison to unmanipulated control animals. It has previously been shown that marking *Desmognathus* salamanders with VIE does not lead to increased noradrenaline or adrenaline in comparison to handling alone (Kinkead *et al.*, 2006), but that handling leads to an increase in glucocorticoid concentrations above baseline levels in blood or urine from a range of amphibian species (Woodley and Lacy, 2010; Narayan *et al.*, 2011b, 2012a, b, 2013). There is evidence of a small increase in faecal glucocorticoid metabolite concentrations from all frogs in this study after marking or handling (Fig. 1), although the individuals used in this study were captive bred and so may have been accustomed to some degree of manipulation. There was an overall decrease in faecal glucocorticoid metabolite concentrations across all frogs over the course of the study, with significantly lower concentrations at the end of the study compared with the other time points (Fig. 2). This may be due to the frogs becoming more familiar with the new environment or individual housing over the course of the study. Cikanek *et al.* (2014) found similar results in Panamanian harlequin frogs (*Atelopus certus* and *Atelopus glyphus*), with frogs maintained individually exhibiting lower faecal glucocorticoid metabolite concentrations than those maintained in groups of two or eight, and those maintained in group housing exhibiting a decline in glucocorticoid metabolite concentrations over the 4 week study period.

Faecal samples have been used to measure glucocorticoid metabolite concentrations in a range of taxa (e.g. Wasser *et al.*, 2000; Bamberg *et al.*, 2001; Martínez-Mota *et al.*, 2008; Santymire *et al.*, 2012; Smith *et al.*, 2012), including amphibians (Cikanek *et al.*, 2014). The values for faecal glucocorticoid metabolite concentrations obtained in the study presented here are similar to those obtained from *A. certus* and *A. glyphus*, and adrenocorticotrophic hormone challenges in these species indicated a similar lag time in glucocorticoid metabolite deposition in the faeces as for *A. callidryas* in this study (Cikanek *et al.*, 2014). Changes in glucocorticoids are observed much more rapidly in urine and blood (within minutes to 2 days; e.g. Woodley and Lacy, 2010; Narayan *et al.*, 2011a, b, 2012a, 2013; Graham *et al.*, 2013). Faecal sampling, however, offers an almost completely non-invasive method for monitoring adrenal activity in comparison to blood or urine collection, which requires handling of individuals to collect samples and thus can confound the data. Moreover, there is evidence from studies with mammals that faecal glucocorticoid metabolites remain relatively stable over a number of days at room temperature (Washburn and Millspaugh, 2002; Evans *et al.*, 2013). The suitability of faecal or urine sampling for different species will be dependent on how a given species separates the deposition of glucocorticoid metabolites in waste products (e.g. Wasser *et al.*, 2000; Bamberg *et al.*, 2001). For example, attempts to validate glucocorticoid metabolites biochemically in the urine of *A. callidryas* and *Agalychnis moreletii* by our research group were unsuccessful (R. Purcell, personal observation), indicating that these species may primarily deposit glucocorticoid metabolites in their faeces.

Overall, there was a decrease in the abundance of bacteria on the skin of all frogs throughout the study period in both treatment groups, which may be related to the change in environment for the frogs (from group housing in naturalistic vivaria to individual housing in more sterile experimental enclosures). Küng *et al.* (2014) also observed shifts in microbial communities over time, and environmental conditions in captivity are known to affect the bacterial communities associated with *A. callidryas* (Loudon *et al.*, 2013; Michaels *et al.*, 2014). However, at the end of study the marked frogs had a significantly greater bacterial abundance than the control frogs, with an elevated fungal abundance in comparison to themselves post-marking. Although the variation around the fungal abundance data for marked frogs at the end of the study is considerable (Fig. 4), fungi were cultured from four of eight frogs in the marked group at this time point, in comparison to one frog post-marking and no frogs at the start of the study. These results indicate there may be some delayed microbial growth on the skin of frogs in response to VIE marking, although it is not clear if this continued beyond the end of the study or whether the bacterial communities had undergone a proliferation between the post-marking and end-of-study time points, and the results seen at the end of the study were the microbial communities returning to normal. However, no obvious skin infections were observed in frogs for 2 weeks after the study had finished.

The cause of the delayed greater abundance of microbes (although minor) on the skin of marked frogs is unclear. The VIE tags were unlikely to be completely sterile due to the preparation methods, and the greater abundance of microbes may reflect a minor infection from the tags. Alternatively, it is possible that the act of marking caused a disruption in the dynamics of the bacterial communities that may have had delayed effects for the microbial community or elicited a physiological response in the frogs. For example, studies in rats and humans have shown increases in immunoglobulins, lymphocytes and macrophages in response to injection of silicone (as contained in VIE tags; Smalley *et al.*, 1995; Hill *et al.*, 1996). The implications of the greater microbial abundance is not known, and it would be of interest to investigate the susceptibility of amphibians to *B. dendrobatidis* after tagging with VIE and other marking systems. Moreover, culturing methods are known to underestimate microbial diversity greatly (reviewed by Amann *et al.*, 1995), and molecular techniques (e.g. next-generation sequencing) are required to characterize the community more fully and determine whether VIE marking and other techniques affect the non-culturable portion of the microbiome.

The results presented here are in contrast to the results found by Antwis *et al.* (2014a), which demonstrated a major and rapid proliferation in culturable bacteria on the skin of *A. moreletii* 1 day after marking with PIT tags. The needle used for VIE tagging is much narrower (30 gauge or 0.31 mm outer diameter) than for PIT tagging (18 gauge or 1.27 mm), and the ‘foreign body’ inserted under the skin is much smaller; therefore, frogs undergoing PIT tagging may experience greater adrenal activity than those that are VIE tagged, leading to differences in the responses of the microbial community (Simmaco *et al.*, 1997, 1998; Antwis *et al.*, 2014a). However, definitive conclusions are difficult to draw because the two studies were conducted on different *Agalychnis* species and the restraint time was slightly longer during PIT tagging than VIE marking (∼1 minute and 30 s, respectively).

There were no changes in the quantity of peptides released by frogs throughout the study period for either treatment group, indicating that VIE marking has no effect on availability or release of peptide stores of *A. callidryas*. This result is expected given that VIE marking also had no effect of faecal glucocorticoid metabolite concentrations, which have the potential to inhibit the production of new peptides for storage (Simmaco *et al.*, 1997, 1998). To our knowledge, this is the first study to quantify the effects of a marking technique on the production of peptides by amphibians, and further research into the effects of other marking techniques (particularly PIT tagging) on peptide production and release is required.

Overall, we have shown that there is no effect of VIE marking on adrenal response (represented by faecal glucocorticoid metabolite concentrations) or peptide release of *A. callidryas*, although there was evidence of a minor increase in microbial abundance on the skin. This indicates that VIE may be a preferable marking technique to PIT tagging (which causes a rapid and major proliferation of skin microbes), particularly in the context of probiotic trials or time course studies that investigate symbiotic bacterial communities. Although more work is required to determine differences between host species in response to different marking techniques, as well as the effects of marking on the susceptibility of amphibians to disease, these results together with those of Antwis *et al.* (2014a) indicate that captive amphibians should not be released immediately after marking, and that where possible, probiotic treatments should not be applied to individuals less than 3 or 4 weeks after marking. Moreover, marking of wild amphibians should be carefully considered.

## Supplementary material

Supplementary material is available at *Conservation Physiology* online.

## Funding

This work was supported by a Biotechnology and Biological Sciences Research Council (BBSRC) PhD studentship to R.E.A.

## Supplementary Material

Supplementary Data
