# Supplementary material for: Effects of visible implanted elastomer marking on physiological traits of frogs
Source: Conserv Physiol. 2014 Oct 3;2(1):cou042. doi: 10.1093/conphys/cou042 (PMC4732488; doi:10.1093/conphys/cou042)
Supplement: Supplementary Data [file supp_2_1_cou042__index.html]

Supplementary Data 

# Effects of visible implanted elastomer marking on physiological traits of frogs

## Supplementary Data

Supplementary Data

**Files in this Data Supplement:**

- Supplementary Data - Docx file
