# Supplementary material for: Effects of visible implanted elastomer marking on physiological traits of frogs
Source: Conserv Physiol. 2014 Oct 3;2(1):cou042. doi: 10.1093/conphys/cou042 (PMC4732488; doi:10.1093/conphys/cou042)
Supplement: Supplementary Data [file supp_cou042_cou042supp.docx]

**Table S1**

Results of posthoc pairwise comparisons from the generalised linear mixed model analyses for effects of visible implant elastomer marking on physiological traits of *Agalychnis callidryas*. An * indicates a statistically significant result (p < 0.05).

| Pairwise comparison group 1 | Pairwise comparison group 2 | Faecal glucocorticoid metabolite concentration | Bacterial abundance | Fungal abundance | Peptide concentration |
| --- | --- | --- | --- | --- | --- |
| Control frogs, start of study | Marked frogs, start of study | 0.709 | 0.988 | 1.000 | 0.990 |
| Control frogs, start of study | Control frogs, post marking | 0.948 | <0.001 * | 0.998 | 1.000 |
| Control frogs, start of study | Marked frogs, post marking | 0.992 | 0.734 | 0.996 | 1.000 |
| Control frogs, start of study | Control frogs, end of study | 0.002 * | <0.001 * | 0.996 | 0.542 |
| Control frogs, start of study | Marked frogs, end of study | 0.097 | 0.604 | 0.995 | 1.000 |
| Control frogs, post marking | Marked frogs, post marking | 0.856 | 1.000 | 1.000 | 1.000 |
| Control frogs, post marking | Control frogs, end of study | 0.001 * | <0.001 * | 1.000 | 0.474 |
| Control frogs, post marking | Marked frogs, end of study | 0.032 * | 1.000 | 1.000 | 1.000 |
| Control frogs, end of study | Marked frogs, end of study | 1.000 | 0.001* | 0.863 | 0.898 |
| Marked frogs, start of study | Control frogs, post marking | 0.370 | 0.207 | 0.999 | 0.991 |
| Marked frogs, start of study | Marked frogs, post marking | 0.946 | <0.001 * | 0.999 | 0.983 |
| Marked frogs, start of study | Control frogs, end of study | 0.802 | <0.001 * | 0.999 | 0.980 |
| Marked frogs, start of study | Marked frogs, end of study | 0.312 | <0.001 * | 0.999 | 0.998 |
| Marked frogs, post marking | Control frogs, end of study | 0.489 | <0.001 * | 1.000 | 0.811 |
| Marked frogs, post marking | Marked frogs, end of study | 0.152 | <0.001 * | <0.001 * | 1.000 |
